# Community Geriatrics

**Published:** 1973-07

**Authors:** G. R. Burston

**Affiliations:** Consultant Geriatrician, Bristol


### Community Geriatrics

G. R. BURSTON, M.B., Ch.B., M.R.C.P.
Consultant Geriatrician, Bristol

### INTRODUCTION

In 1971 negotiations were started with various General Practitioners and Medical Officers of Health to investigate the possibility of establishing regular assessment clinics in various Health Centres for elderly patients in the geriatric department's catchment zone. The Southmead Geriatric Department catchment area is some 30 miles long stretching from Berkeley to Clevedon and some five miles wide from the Bristol Channel to about the line of the A38 road. There are approximately 36,000 elderly persons (65 years and over) residing in it, the majority in the Bristol County (23,000 approx.), 5,000 in rural South Gloucestershire and 8,000 in rural North Somerset. There are four Health Centres in this zone; at Clevedon (with the highest incidence of elderly, about 23 per cent of population), Southmead, Nailsea and Thornbury. Three Health Centres decided to participate.

The clinics are held regularly once monthly in the afternoon so that forward planning can take place. The general organisation of the clinic is done by the Health Centre Supervisor who arranges the bookings and notifies the Geriatric Department secretary of the number attending and basic problems involved and supplies information about previous hospital attendances or admissions so that old records may be obtained and scrutinised for relevant information before the clinic. She also arranges transport to bring the patients to the clinic. Stretcher patients are not brought to the clinic as, by their degree of infirmity, they are unsuitable. Wheelchair patients are accepted.

### METHOD (FIRST YEAR REVIEW 1972/73)

The clinic is conducted by the Consultant Geriatrician; four new patients are seen and carefully selected follow-up cases may be reviewed at the discretion of the Consultant or the General Practitioner. Occasionally follow-up of a patient living locally who had previously been in hospital is arranged at the clinic for convenience. Most patients are visited before the clinic either by a Health Visitor (Southmead and Thornbury Clinic) or a District Nurse (Nailsea) who takes routine blood samples on her visit. Sometimes these visits are used to introduce the patient to the idea of the purpose of the clinic visit as well as using her short visit to have an up-to-date assessment of the domestic situation and applying, in certain cases, a mental assessment test to the patient.

The Somerset Social Services Department have co-operated to the full with the Nailsea Clinics by supplying a Social Worker for each session who sees the patient and/or relatives to ensure that the patient is receiving such benefits as are due and also to enquire about the domestic situation to see if further help can be given. The Social Worker will help in the mental assessing of the patient in the clinic if the form has not been previously completed and will contribute to the discussion at the end of the clinic. The Geriatrician is assisted by the District Nurse who visits the patient at home in Nailsea, the Geriatric Deparament part-time Health Visitor in the Southmead Clinic and a local Health Visitor or District Nurse at Thornbury.

At some stage nine General Practitioners have called into the clinic either during the session or afterwards to discuss their patients. Other visitors to the clinic have included a medical student, a General Practitioner Vocational Trainee, the Geriatric Department Registrar (who has assisted twice), various local district nurses and health visitors, community workers and a home help.

Each patient is allocated 40 minutes of medical time with the rest of the time spent in the clinic being taken up by the other members of the team assessing and discussing various problems with the patient and relatives. Most visits last between 75-100 minutes before the patient is transported home. Two of the clinics regularly supply the patient with a cup of tea before leaving.

In the Southmead and Thornbury Clinics all specimens for the laboratory are collected in time for transport to reach the Pathology Department for appropriate handling before the department closes. The Southmead Pathology Service has been most helpful in this way. Two of the Health Centres have electrocardiographs and a 10-12 lead E.C.G. is taken on most patients during their visit. A specimen of urine is tested wherever possible with 'Labstix' and if unobtainable but deemed necessary arrangements are made for a sample to be collected at home and tested. The patients are weighed. Dressing gowns are now provided in all clinics. Careful observations of the patient's ability to carry out simple daily living functions whilst in the clinic are made and recorded.

### RESULTS

Twenty two General Practitioners use the three Health Centres. During the first 10 months of the Geriatric Clinics 20 (91 per cent) of these doctors referred patients to the clinics.

The sex distribution of patients at the Nailsea and Thornbury Clinics was equal whereas the ratio male/female in the Southmead Clinic was 1:2.5, which is the generally accepted ratio of demand for the Geriatric in-patient service. The age of these clinic patients ranged from 66 years to 92 years, the majority (75 per cent) being between 75-84 years old. The average age for the patients was 77.9 years which is lower than the average (82.2 years) for in-patients.

All patients had a comprehensive letter from the family doctor; some of these were quite outstanding

| | Southmead | Thornbury | Nailsea | Total |
|---|---|---|---|---|
| Possible Sessions | 10 | 10 | 8 | 28 |
| Actual Sessions | 9 | 6 | 7 | 22 (82%) |
| Possible New Cases | 36 | 24 | 28 | 88 |
| Actual New Cases | 35 | 22 | 24 | 81 (99%) |
| Follow-up Cases | — | 3 | 3 | — |

**Table 1 Possible Use of Clinics related to actual use.**

in their quality, my impression being that they were far better than usually found in a hospital out-patient clinic. On numerous occasions the local Health Visitor had provided additional written information. Perhaps the most commonly neglected area in the pre-clinic work-up was that of the elderly person's mental state particularly with reference to depressive illness. Drug prescribing information was often poorly documented in the referring letters and frequently did not coincide with the patient's version. Complete physical examination was performed and all patients had a rectal examination performed but vaginal examinations were beyond the scope of this clinic.

As would be expected significant physical and, more rarely, mental illness was detected, illness which had not apparently been appreciated by the doctor before the clinic. Several practitioners commented on the fact that after their first patient had visited the clinic they had spent extra time examining their subsequent patients before the next clinic so as to 'not miss too much' as one put it but there was no evidence to suggest that this practice continued as there was a feeling that 'to find this pathology was the whole point of the clinic'.

The table of signficant illness found but apparently not suspected may contain some diseases the family doctor knew about although failed to refer to in his letter of introduction about the patient.

Nothing 'new' was discovered in only five patients; 35 patients contributed most of the pathology found in Table II and in the majority of the remainder some additional minor pathology was demonstrated and advice given about its subsequent management. One does not know the degree of selectivity of these patients by the family practitioners concerned but as this clinic was initially established as one for elderly patients thought to be at some particular risk it is reasonable to assume that the finding of significant pathology in 40 per cent of the patients could be expected. Referral to the clinics came in some cases initially from District Nurses or Health Visitors to the family practitioners—the patient not being previously known to the general practitioner.

Geriatricians have recognised for a long while that multiple pathology is the order of the day when dealing with the elderly; most old people have at least two potentially incapacitating diseases present. Some attempt was made to determine the number of pathological conditions in these patients.

An extreme example of multiple pathology in an elderly patient was seen in the case of one 84 year old man who was found to have:

- Total blindness (but not registered or known to Social Services)
- Ischaemic heart disease, atrial fibrillation, uncontrolled congestive heart failure, peripheral arterial disease, ischaemic leg ulcers.
- Generalised active arthritis involving both large and small joints.
- Prostatism and bilateral inguinal herniae.
- Deafness.
- Social problems.
- Reactive depression with suicidal tendencies.

| | Number | | Number |
|---|---|---|---|
| **Carcinoma** | | **Endocrine disorders, and blood dyscrasias** | |
| Site | Number | Hypothyroidism | 3 |
| Stomach | 1 | Pernicious anaemia | 1 |
| Rectum | 1 | Diabetes Mellitus | 1 |
| Breast | 1 | Scurvy | 1 |
| Lung (+ 2 suspect) | 1 | **Cardiovascular Disease** | |
| **Cerebrovascular Disease** | | Silent Myocardial infarction | 3 |
| Unrecognised strokes | 2 | Essential Hypertension (with diastolic pressure greater than 130 mmHg.) | 2 |
| Carotid artery stenosis with transient strokes | 1 | Postural Hypotension (with systolic pressure fall of more than 20 mmHg on rising) | 4 |
| **Alimentary disease** | | **Other conditions** | |
| Peptic ulceration with anaemia | 2 | Unrecognised Parkinson's disease | 1 |
| Bleeding haemorrhoids | 1 | Severe depression | 2 |
| Malabsorption syndrome | 1 | Osteomalacia | 1 |
| Cholecystitis and cholelithiasis | 1 | Severe visual loss | 2 |
| **Inappropriate medication** | | | |
| Overdigitalisation | 4 | Inappropriate diuretic | 1 |
| Do. and with hypokalaemia | 2 | | |

**Table 2 Apparently unrecognised but significant disease found in clinic patients**

| No major disorder | 1 maj. | 1 maj./1 min. | 1 maj./ 2+ min. | 2 maj./1 min. | 2 maj./2 min. | |
|---|---|---|---|---|---|---|
| 4.5% | 12% | 16% | 23.5% | 11% | 7% | Female |
| — | 34% | 4% | 25% | — | 12.5% | Male |
| 2 maj/3 min. | 2 maj/3 min+ | 3 maj/1 min. | 3 maj/2 min. | 3 maj/3 min+ | | |
| 7% | — | 2.5% | 9.5% | 7% | | Female |
| — | 4% | — | 8% | 12.5% | | Male |

**Table 3 Different disease/pathology present in the patients**

### OUTCOME OF THE VISIT AND SUBSEQUENT ACTION

Eight patients were sufficiently ill at the time or needed urgent further investigation to be admitted within 24 hours to the nearest Geriatric Unit. One of these patients died, the remainder were discharged successfully back to the community and have not needed re-admission to the Department. A further six have been admitted subsequently to the Department with an exacerbation of one of their major diseases; one has died; two have been discharged and three are still in hospital. One patient, referred urgently to the Social Services, failed to obtain a place in a residential home and has now unfortunately become permanently hospitalised. Two patients, each with varying degrees of dementia, have had short periods of holiday relief within the Department; one is now living with her family rather than on her own and one is still waiting for admission to residential care some nine months after being referred to the local Social Services. Two patients, both male, have needed in-patient care in Psychiatric Hospitals because of exacerbations of significant dementia, one not recognised until he originally attended the clinic. One other patient had a short period in a general medical unit following a suspected haematemesis. Four patients needed urgent surgical attention and a further four were referred to other specialties for their expert help. Seven patients have attended the local Geriatric Unit as day patients for a further assessment/investigation and treatment but none have required admission to that unit. Two patients are known to have died shortly after attending the clinic, both as the result of conditions recognised at the clinic. In all, 17 patients were thought to be in need of residential care in a Social Services home; 12 in an ordinary home and 5 in a home for the elderly mentally infirm. Four of these patients, who have received in-patient treatment since seen in the clinic, would not have required hospitalisation if they could have gained admission at an earlier stage to Part III accommodation.

### THOUGHTS

The establishment and continuation of such clinics as described above can only be maintained if they serve a useful purpose. They consume much specialist medical time and may occupy up to four hours of District Nurses' time before and during the clinic or similar time for at least one health visitor and/or social worker, perhaps longer for these two groups if follow-up investigation and help are required as a result of the clinic. They contributed to the work load of the pathology service and the cost of the investigations probably amounts to about £1 per head. The organisation of the clinic and subsequent production of a comprehensive report is also time-consuming and adds to the overall cost of the service. A rough estimate would suggest that each patient costs about £5 for all personnel and labour. There is, however, saving in transport cost by use of private cars or hospital car type service. It is impossible to estimate the intrinsic value to the patient and relative. So far no complaint has been received from either source whereas considerable satisfaction has been expressed at the end of the session by many of the patients. They appear to appreciate the more normal surroundings, the informal atmosphere and particularly the reassurance that often something can be done despite the gloom their state has precipitated in themselves and others. The service is certainly cheaper than a formal domiciliary consultation especially if an E.C.G. is performed or any other minor procedure carried out at the patient's home.

These clinics have demonstrated that many unrecognised pathological conditions can be found in elderly people attending a specialist clinic based on a Health Centre. Such clinics not only appear to stimulate interest in the elderly in the locality among doctors, nurses, health visitors and social workers but also seem to allow potentially dangerous conditions to be treated or corrected in many of the patients. The clinics give the opportunity for assisting the old person or his family to plan comprehensively for the future. This in turn protects the patient, protects the practitioner and protects the hospital bed.

